# *ProtParCon*: A Framework for Processing Molecular Data and Identifying Parallel and Convergent Amino Acid Replacements

**DOI:** 10.3390/genes10030181

**Published:** 2019-02-26

**Authors:** Fei Yuan, Hoa Nguyen, Dan Graur

**Affiliations:** Department of Biology and Biochemistry, University of Houston, Houston, TX 77204, USA; fyuan4@uh.edu (F.Y.); hhnguyen5@uh.edu (H.N.)

**Keywords:** bioinformatics pipeline, parallelism, convergence, adaptation

## Abstract

Studying parallel and convergent amino acid replacements in protein evolution is frequently used to assess adaptive evolution at the molecular level. Identifying parallel and convergent replacements involves multiple steps and computational routines, such as multiple sequence alignment, phylogenetic tree inference, ancestral state reconstruction, topology tests, and simulation of sequence evolution. Here, we present *ProtParCon*, a Python 3 package that provides a common interface for users to process molecular data and identify parallel and convergent amino acid replacements in orthologous protein sequences. By integrating several widely used programs for computational biology, *ProtParCon* implements general functions for handling multiple sequence alignment, ancestral-state reconstruction, maximum-likelihood phylogenetic tree inference, and sequence simulation. *ProtParCon* also contains a built-in pipeline that automates all these sequential steps, and enables quick identification of observed and expected parallel and convergent amino acid replacements under different evolutionary assumptions. The most up-to-date version of *ProtParCon*, including scripts containing user tutorials, the full API reference and documentation are publicly and freely available under an open source MIT License via GitHub. The latest stable release is also available on PyPI (the Python Package Index).

## 1. Introduction

Understanding adaptive evolution at the molecular level is a fundamental goal of evolutionary biology [1,2]. One approach to studying adaptive evolution involves the identification of parallel and convergent amino acid replacements in proteins [3]. Identifying parallel and convergent amino acid replacements involves multiple steps and multiple computational routines (e.g., multiple sequence alignment, phylogenetic tree inference, ancestral state reconstruction, topology tests, and sequence simulations). Although specialized resources exist for each step, no integrated tool or framework can be found in the literature for efficient molecular data processing and fast identification of parallel and convergent replacements.

Here, we introduce a Python 3 package called *ProtParCon* (a portmanteau for *Prot*ein *Par*allelism and *Con*vergence) that integrates previously available tools as well as new modules to automate the identification of parallel and convergent amino acid replacements without user intervention. The first step in *ProtParCon* entails the retrieval of one-to-one orthologous protein sequences from the species of interest. Next, it performs a multiple-sequence alignment for all the ortholog sets, followed by ancestral reconstructions using a maximum likelihood approach. Subsequently, we iterate over every protein site in the alignment and identify all parallel and convergent amino acid replacements in all independent branch pairs. Our package automates all these steps within a pipeline for fast and easy parallel and convergent amino acid replacements identification. *ProtParCon* also allows biologists to perform routine tasks easily and quickly using simple functions and commands that do not require any programming skills. The modular design of the package also allows users with even modest programming skills to add more features to the package.

## 2. Description of the Computational Steps

**Figure 1:**
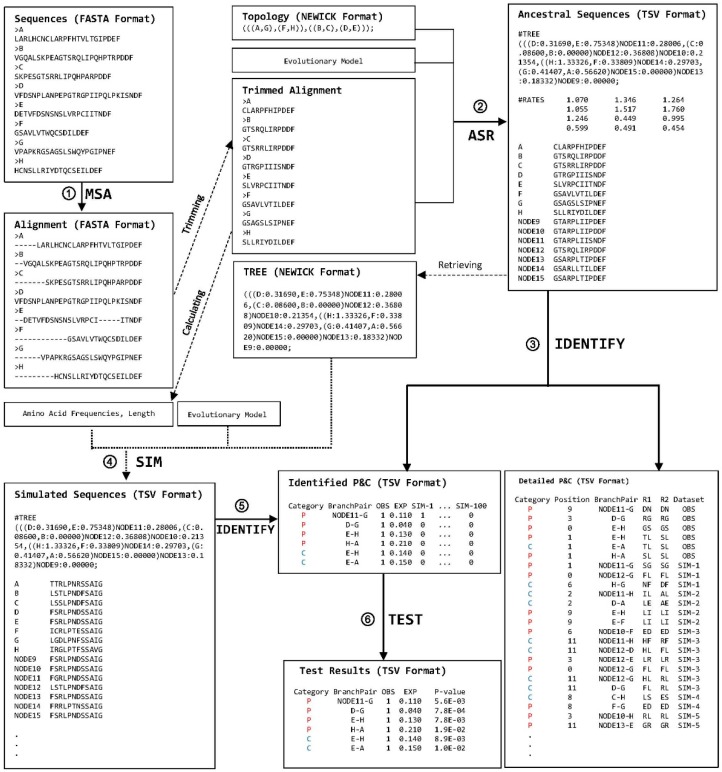
Overview of the *ProtParCon* analytical scheme. During the multiple sequence alignment (MSA) stage, protein sequences are aligned while gaps and ambiguous character states are trimmed. In the ancestral state reconstruction (ASR) stage, ancestral character states at each site are inferred for each internal node in the reconstructed tree. Observed parallel and convergent amino acid replacements for pairs of branches are identified in the IDENTIFY stage. Parallel replacements are denoted by P (red) and convergent replacements by C (blue). Simulations are conducted in the SIM (simulation) stage. Simulated sequences are evolved according to the following parameters: (1) an evolutionary model (a replacement rate matrix), (2) the branching pattern and branch lengths of the tree estimated in the ASR stage, and (3) amino acid frequencies and sequence length estimated from the trimmed alignment. Expected parallel and convergent replacements are identified after the SIM stage or they are directly calculated if no simulation is conducted. The differences between numbers of observed and expected parallel and convergent replacements for branch pairs of interest are tested during the TEST stage. For better readability, only part of the simulated sequences and detailed P&C data are shown. TSV (Tab separated values) format data are reformatted. Notation of branch pair, A-B, means a branch pair involving two branches that are leading to A and B, respectively. R1 and R2 represent two amino acid replacement events along two branches. The standard one-letter abbreviations for amino acids [4] is used for the replacements.

*ProtParCon* processes a set of orthologous protein sequences with known phylogenetic relationships in six stages: MSA, ASR, IDENTIFY, SIM, IDENTIFY, and TEST (Figure 1).

**Figure 2:**
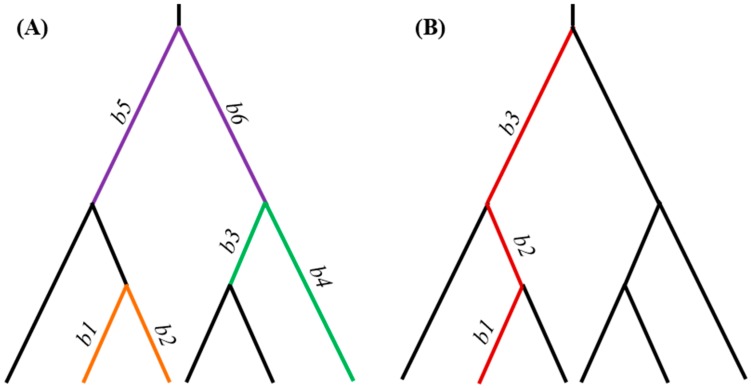
Types of branch pairs that are excluded from comparisons. (**A**) Neighboring branch pairs (e.g., b1-b2, b3-b4, and b5-b6) are excluded from the analysis because it is not possible to infer parallel and convergent substitutions in these pairs. Other such branch pairs exist. (**B**) An example of three branches sharing the same evolutionary path is shown. Branch pairs b1-b2, b1-b3, and b2-b3 are excluded from the analysis because they violate the independent-evolution requirement. Other such branch pairs exist.

Protein sequences in FASTA format are used in the multiple sequence alignment (MSA) stage. The user can select from a selection of multiple sequence alignment programs. Gaps and ambiguous characters are removed from the alignment. The alignment is then passed onto an ancestral state reconstruction program in the ancestral state reconstruction (ASR) stage. A phylogenetic tree in Newick format is used. Users can select a reconstruction program and specify an evolutionary model as well as assign model parameters. After the ancestral states are inferred, *ProtParCon* examines the amino acid replacements along each lineage and identifies parallel and convergent amino acid replacements along amino acid sites for pairs of branches during the IDENTIFY stage. At any given site, parallel replacements are identified as replacements from the same ancestral amino acid to the same descendant amino acid along independent evolutionary lineages. Independent replacements that occurred from different ancestral amino acids to the same descendant amino acid are identified as convergent replacements. Since it is not possible to infer parallel and convergent amino acid replacements in connected branches (i.e., branches descended from the same ancestor), neighboring branch pairs are excluded (Figure 2A). Branch pairs sharing an evolutionary path are also excluded because they violate the independent-evolution requirement (Figure 2B).

Users may also estimate the numbers of expected parallel and convergent replacements under a specific evolutionary scenario. During the SIM stage, the expected numbers can either be estimated by using simulated sequences or calculated by using a method developed in the previous study [5]. The calculation in [5] employs an amino acid replacement probability matrix, site replacement rates, and the branch lengths of the phylogenetic tree.

Statistical tests in the TEST stage determine whether the observed numbers of parallel and convergent replacements can be said to be significantly different from the expectations estimated using the specified null model. The default statistical test in TEST assumes that the number of parallel or convergent sites between two lineages follows a Poisson distribution, with a mean equal to the expected number [5]. When the observed number is smaller than the expected number, the lower tail probability is given; when the observed number is greater than the expected number, the upper tail probability is given.

### 2.1. Implementation of ProtParCon

*ProtParCon* is written in Python 3. The package provides functionalities for processing molecular data by integrating several programs into a common interface. A list of functions and supported programs provided in Table 1. Links to the supported programs, the tested versions, and download/install instructions are available in Table S1.

*ProtParCon* also provides a command line interface. It consists of a set of commands that can be easily used in a terminal (Windows PowerShell or Unix-like shell). The package redefined parameters for the supported programs and simplified the command line arguments, so users can use simpler functions and commands to perform routine tasks. It also redefined outputs, warnings, and errors from the supported programs. The package implemented default parameters for all the supported programs in this release and it will add supports for additional parameters for supported programs in the next release. A full list of all available functions, commands, and their usages are given in the online documentation (https://ibiology.github.io/ProtParCon/). In order to enable experienced users to introduce new methods or software, the source code is hosted on GitHub (https://github.com/iBiology/ProtParCon). Users can also obtain the package from PyPI (https://pypi.org/project/protparcon/).

### 2.2. Package Validation

We validated the accuracy of *ProtParCon* by applying it to the 6,400 orthologous genes from the nine eutherian species in [17], and we were able to obtain the same results as those in their Figure 1.

## 3. Case Study: Parallel and Convergent Amino Acid Replacements in Lysozyme C Sequences

In mammals and birds, lysozyme c is an enzyme that is usually expressed in body fluids (e.g., saliva, serum, tears, avian egg white, and mammalian milk) to defend against invading bacteria [18,19]. In foregut fermenters, such as the ruminants (e.g., cows, deer, sheep, and giraffes), colobine monkeys (e.g., langurs), and hoatzin (a bird most probably related to the cuckoo), lysozyme c is also secreted in the posterior parts of the digestive system and is used to degrade the walls of the bacteria that carry on the fermentation in the foregut, thereby freeing nutrients from within the bacterial cells [4]. The recruitment of lysozyme c to function in the stomach has occurred independently in each of these three lineages. Previous studies have identified several amino acid sites that have experienced parallel and convergent replacements [3,18,20]. These replacements were viewed as evidence for adaptation. To test whether this is indeed the case, parallel and convergent amino acid replacements in a set of 26 mammalian and avian lysozyme c precursor sequences were examined.

### 3.1. Data

**Figure 3:**
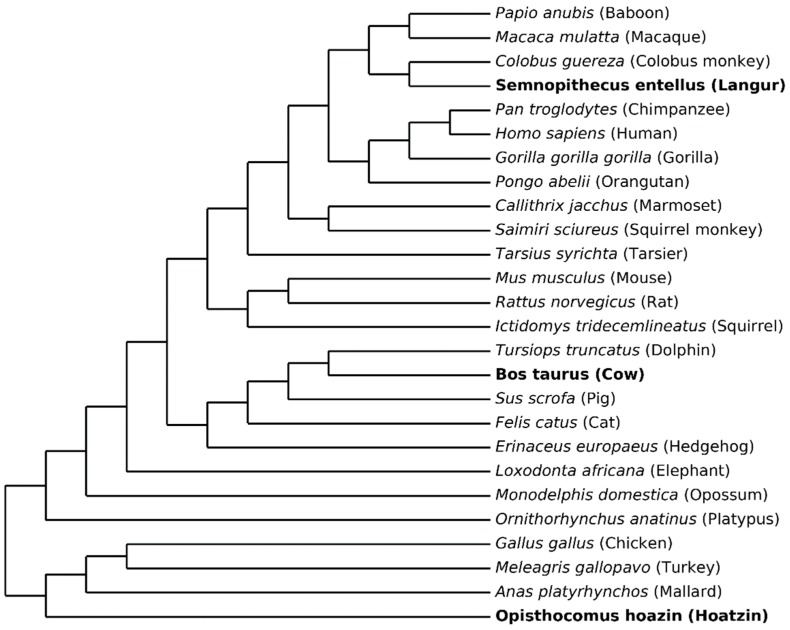
An unscaled tree representing the phylogenetic relationships among 26 mammalian and avian species used in this study. The phylogenetic tree represents a strict consensus tree derived from [22,23,24,25]. Three foregut fermenters are shown in bold.

Twenty-six sequences of lysozyme c precursors were downloaded from the UniProtKB (https://www.uniprot.org/uniprot/?query=job:M201901306746803381A1F0E0DB47453E0216320D1EEE8AE) (The UniProt Consortium, 2017) [21]. Three stomach lysozyme c sequences are from foregut fermenters: langur (*Semnopithecus entellus*), cow (*Bos taurus*), and hoatzin (*Opisthocomus hoatzin*). The topology in Figure 3 was used in both the ancestral state reconstruction and the parallel and convergent replacement identification. With the currently available data, it is difficult to increase the taxonomic representation beyond our 26 species without (1) introducing uncertainties in the species tree, (2) decreasing the length of the aligned sequences, or (3) introducing uncertainties in the one-to-one orthology assignment. This does not mean that the set of sequences cannot be increased, but this requires additional phylogenetic work beyond the scope of this example. For example, we could not use the lysozyme c precursor sequence from horse because the position of Perissodactyla within the eutherian phylogenetic tree has not yet been unambiguously determined.

### 3.2. Parallel and Convergent Amino Acid Replacements in Lysozyme c

In this section, we illustrate the usage of *ProtParCon* as it is applied to a real biological dataset, and discuss the functionalities of the package and its outputs.

As mentioned previously, *ProtParCon* only accepts protein sequences in FASTA format and phylogenetic trees in Newick format. The 26 lysozyme c sequences were saved to a FASTA format file named “lysozyme.fasta.” The species tree topology was saved to a file named “lysozyme.newick.” In the following example, code lines indicated with “>>>” are within the Python IDE console, but can equally be incorporated into standalone scripts.

The sequence alignment was done in *ProtParCon* via these two commands:

>>> from ProtParCon import msa, asr, imc, sim, detect

>>> alignment = msa(“muscle”, “lysozyme.fasta”, trimming=True)

In this case, program MUSCLE was used to align the lysozyme c sequences. Users, of course, can choose any other supported alignment program (Table 1). Since “*trimming*” is set to True, all sites with gaps and ambiguous characters are removed. Function *msa* produces two FASTA format files, one is the alignment computed by MUSCLE and the other is the trimmed alignment. The function also returns the file path of the trimmed alignment file and stores it to variable “*alignment*”. Using this variable, ancestral state reconstruction is done by:

>>> ancestors = asr(“codeml”, alignment, “lysozyme.newick”, “JTT”)

Function *asr* requires an ancestral state reconstruction program (in this case, CODEML), a FASTA format alignment file, a NEWICK format tree file (“lysozyme.newick”), and an evolutionary model (in this case, JTT [26]). Calling this function will generate a tab-delimited text file. The first line is the guide tree in NEWICK format with branch length estimated and internal nodes labeled. The third line contains the tab separated replacement rate for each site. The fifth and all the lines below are ancestral sequences (the original alignment for terminal sequences are also included). Function asr returns the file path of the ancestral sequence file. To identify parallel and convergent amino acid replacements, users just need to pass the ancestral sequence file to function *imc*:

>>> imc(ancestors)

Calling function *imc* produces two tab-delimited text files that contain the main results. One file stores the counts of identified parallel and convergent amino acid replacements for each branch pair and the other stores details of all identified replacements (i.e., type, position, branch pair, and amino acid replacements of an identified event). From these two files, users can get all information about observed parallel and convergent amino acid replacements. To obtain the expected number of replacements, one more line of code is needed:

>>> simulations = sim(“evolver”, model=”selection-free.dat”, anc = ancestors)

Calling function *sim* will simulate sequences using an external program EVOLVER. Amino acid replacement will be introduced according to the selection free model based on the mutation patterns of pseudogenes in human genome (”selection-free.dat”) [27]. All other information for running this simulation (i.e., sequence length, phylogenetic tree with branch length, and amino acid frequencies) will be retrieved from the ancestral sequence file generated earlier. The function produces a tab-delimited text file storing simulated sequences:

>>> imc(simulations)

Here, the function *imc* is called again to calculate the expected numbers of parallel and convergent amino acid replacements using simulated sequences.

To test whether or not the numbers of observed parallel and convergent replacements are significantly different from their chance expectations, the function *detect* is called.

>>> detect()

Without any parameters, this function tests the differences for all branch pairs with a testing method proposed in a previous study [5] and without correcting p-values for multiple comparisons. In practice, users should determine the branch pairs they are interested in and select post-hoc statistical tests and corrections for multiple comparisons on a case-by-case basis.

The five functions (msa, asr, imc, sim, and detect) in *ProtParCon* can be used in a step-by-step manner. However, a more convenient way is to use function *imc* (it has a built-in pipeline that chains all the other four functions together behind the scene):

>>> from ProtParCon import imc, detect

>>> imc(“lysozyme.fasta”, tree=“lysozyme.newick”, aligner=“muscle”,

... ancestor=“codeml”, simulator=“evolver”, asr_model=“JTT”,

... exp_model=“JTT”)

>>> detect()

### 3.3. Results and Tentative Conclusions

Parallel and convergent amino acid replacements for 904 branch pairs in the phylogenetic tree (Figure 3) that are neither connected branches nor branches sharing an evolutionary path were examined along 138 unambiguously aligned amino acid sites. Among these branch pairs, at least one parallel amino acid replacement was found in 188 branch pairs, while at least one convergent amino acid replacement was found in 26 branch pairs. Seventeen branch pairs had both parallel and convergent changes.

**Figure 4:**
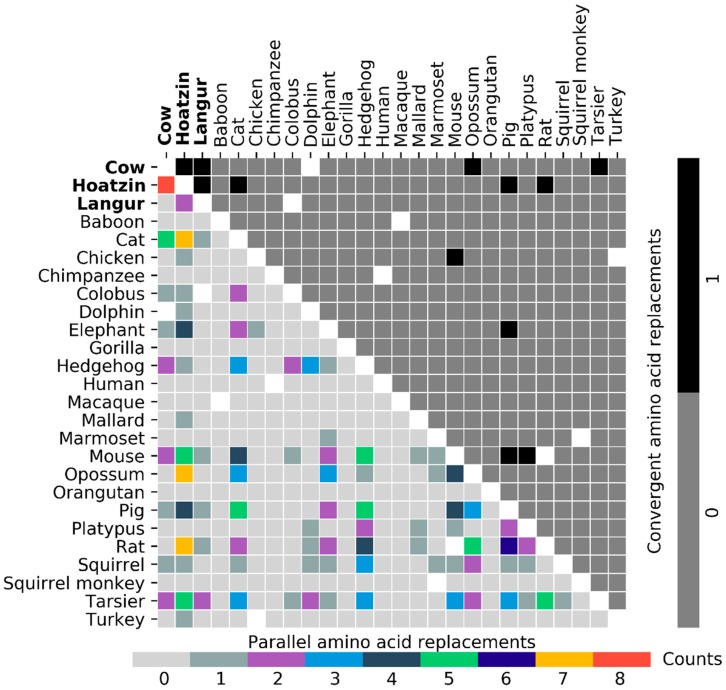
Observed numbers of parallel (lower triangle) and convergent (upper triangle) amino acid replacements for branch pairs involving only terminal branches. Off-diagonal empty squares indicate the branch pairs were excluded from the comparisons due to each of them involving two connected branches. Three foregut fermenters are shown in bold.

Considering only the 318 branch pairs that involve terminal branches, 204 parallel amino acid replacements in total were found for 86 branch pairs, while 13 convergent amino acid replacements in total were found for 12 branch pairs. Nine branch pairs had both parallel and convergent changes. (Figure 4).

As far as foregut fermenters are concerned, parallel replacements were identified in the cow-langur and the cow-hoatzin branch pairs, while convergent replacements were identified in all three possible comparisons. The largest number of parallel replacements between two lysozyme c sequences were found in the hoatzin-cow comparison. However, for nearly half of the comparisons (39 out of 86 comparisons), only one parallel replacement was found.

For all branch pairs involving only terminal branches and having at least one parallel or convergent replacement, we also tested whether or not the number of observed parallel and convergent replacements are significantly different from their chance expectations. The test results are shown in Table 2. We note however, that the *p* values in the Table have not been corrected for multiple comparisons, so the number of statistically significant results is almost certainly grossly overestimated.

Notwithstanding the above caveat, the number of branch pairs in which the observed parallel and convergent changes exceeded the expectation under the selection-free model was quite small (8% and 2% for parallel and convergent replacements, respectively).

In Table 2, we note that a certain number of parallel changes in one case may be statistically significant, while the same number in another case may not be. For example, there is one parallel replacement identified in the squirrel-marmoset comparison and another in the cow-squirrel comparison, but the former is significantly greater (*p* = 0.0462) than the expectation and the latter is not significant (*p* = 0.2569). This may seem unexpected, but it is actually reasonable. Rather than comparing the number of replacements identified in each comparison, the statistical test compares the identified number of parallel or convergent replacements in each case with its random chance expectation. Since the expectations of different comparisons are different, there is no reason that the same number of parallel or convergent replacements identified in different comparisons will give the same statistical test result.

It is beyond the scope of this example to exhaustively investigate the evolution of lysozyme c in foregut fermenters. Here, we can only address the question of whether or not parallel and convergent changes in lysozyme c of cow, langur, and hoatzin can be used to support the claim that in each of these lineages the enzymes adapted to similar conditions through identical changes in the protein sequence. Several facts argue against the claim of adaptation. First, although the number of parallel amino acid replacements in the hoatzin-cow comparison is significantly higher than the expected number, the numbers of parallel replacements in other comparisons, such as hoatzin-cat, hoatzin-rat, and pig-rat, are also significantly higher than the expectations (Table 2). Second, most parallel amino acid replacements were found to involve conservative amino acid replacements. For example, among the eight parallel amino acid replacements in the cow-hoatzin comparison, five involved a change from arginine and lysine—two basic amino acids that are frequently exchanged in the evolution of proteins without any functional consequences. Of course, conservative amino acid replacements may have important functional consequences in particular cases, but on average they usually do not [28]. Third, by comparing the eight sites that experienced parallel amino acid replacements in the hoatzin-cow comparison with two known active sites, eight structurally important sites (https://www.uniprot.org/uniprot/P04421 accessed on November 6, 2018), and 4 sites in which amino acid changes have been shown to cause disease (renal amyloidosis) (https://www.omim.org/entry/153450#0001 accessed on November 6, 2018), we found that none of these sites coincided with the sites that experienced parallel changes. Again, it should be noted that adaptive changes may have nothing to do with these sites. Fourth, despite the fact that lysozyme c in foregut fermenters should be adapted to extremely acidic environments, which would require a change in the charge of the proteins, the parallel and convergent amino acid replacements in foregut fermenters do not exhibit any consistent directionality in charge changes. Out of 24 changes, 22 do not affect charge, two changes are from a basic amino acid to an uncharged one, two are from an acidic amino acid to a basic one, and two are from an uncharged amino acid to an acidic one. Finally, as mentioned above, we made no attempt to correct the *p* values for multiple comparisons.

## 4. Discussion

To the best of our knowledge, in spite of the drastic expansion of public software for computational routines, there exists no framework or pipeline that enables automatic identification of parallel and convergent amino acid replacements. In developing *ProtParCon* we sought to address this deficit. For example, by providing the necessary input parameters to the function *imc*, it will perform multiple sequence alignment, ancestral reconstruction, simulation, and identifying, calculating the observed and expected amino acid replacements. As a case study, we applied *ProtParCon* to identify parallel and convergent replacements in lysozyme c sequences and to test whether or not they occur more than expected by random mutation. We demonstrated that parallel and convergent amino acid replacements can be readily identified based on real biological data.

We note that because of its modular design, users can easily add functionalities to *ProtParCon*. One possible new function could be adding programs that focus on assessing the quality of multiple sequence alignments, for example, [29,30] and removing unreliable columns from the alignment.

## Figures and Tables

**Table 1:** Summary of functions and supported programs in *ProtParCon*.

Functions	Description	Supported Programs
oma	For OMA orthology database	N/A
msa	For multiple sequence alignment	MUSCLE [6] MAFFT [7] Clustal Omega [8] T-COFFEE [9]
asr	For ancestral states reconstruction	CODEML ^1^ RAxML [10]
mlt	For maximum-likelihood tree inference	FastTree [11] IQ-TREE [12] RAxML PhyML [13]
aut	For topology test ^2^	IQ-TREE
sim	For protein sequence simulation	EVOLVER ^1^ Seq-Gen [14]
imc	P&C identification ^3^	N/A

^1^ CODEML and EVOLVER are implemented in PAML package [15]. ^2^ Only the approximately unbiased test [16] is supported. ^3^ A built-in pipeline for parallel and convergent amino acid replacement identification.

**Table 2:** Tests of parallel and convergent evolution of lysozyme c sequences. Each of the three branch pairs leading to two foregut fermenters is shown in bold. All other comparisons involving two terminal branches with either at least one parallel replacement or at least one convergent replacement are also listed. A statistical test is performed under the assumption that the number of parallel (or convergent) replacements for a given branch pair follows a Poisson distribution with the mean equal to the expected number. When the observed number is smaller than the expected, the lower tail is given; otherwise, the upper tail probability is given. Significant difference between the observed and expected numbers is marked with * (*p* ≤ 0.05) or ** (*p* ≤ 0.01). If the observed and the expected numbers of replacements in both categories are 0, then *p* cannot be calculated and the *p* value is marked with N/A.

Branch Pair	Parallel Replacement			Convergent Replacement		
	Obs.	Exp.	*p*-Value	Obs.	Exp.	*p*-Value
**Cow-Langur**	0	0.43	0.6505	1	0.35	0.0487 *
**Cow-Hoatzin**	8	1.51	0.0000 **	1	0.78	0.184
**Langur-Hoatzin**	2	0.52	0.0159 *	1	0.32	0.0415 *
Cow-Squirrel	1	0.98	0.2569	0	0.23	0.7945
Cow-Mouse	2	1.96	0.3125	0	0.57	0.5655
Cow-Tarsier	2	1.51	0.1937	1	0.5	0.0902
Cow-Colobus	1	0.34	0.0462 *	0	0.17	0.8437
Hoatzin-Mallard	1	0.61	0.1252	0	0.43	0.6505
Hoatzin-Chicken	1	0.35	0.0487 *	0	0.27	0.7634
Hoatzin-Turkey	1	0.34	0.0462 *	0	0.23	0.7945
Hoatzin-Opossum	7	3.08	0.0137 *	0	0.14	0.8694
Hoatzin-Elephant	4	1.43	0.0155 *	0	0.26	0.7711
Hoatzin-Hedgehog	1	1.6	0.5249	0	0.46	0.6313
Hoatzin-Cat	7	1.98	0.0010 **	1	0.73	0.1663
Hoatzin-Pig	4	2.1	0.0621	2	0.64	0.0273 *
Hoatzin-Dolphin	1	0.94	0.2422	0	0.42	0.657
Hoatzin-Squirrel	1	1.22	0.6554	0	0.35	0.7047
Hoatzin-Mouse	5	2.62	0.0505	0	0.72	0.4868
Hoatzin-Rat	7	1.88	0.0007 **	1	0.59	0.1186
Hoatzin-Tarsier	5	2.02	0.0173 *	0	0.74	0.4771
Hoatzin-Colobus	1	0.38	0.0563	0	0.28	0.7558
Langur-Mouse	1	0.57	0.1121	0	0.28	0.7558
Langur-Rat	1	0.55	0.1057	0	0.3	0.7408
Langur-Tarsier	2	0.48	0.0129 *	0	0.27	0.7634
Langur-Pig	1	0.46	0.0783	0	0.29	0.7483
Langur-Cat	1	0.38	0.0563	0	0.27	0.7634
Cat-Pig	5	2.87	0.0714	0	0.00	N/A
Cat-Cow	5	1.5	0.0045 **	0	0.42	0.657
Cat-Squirrel	1	1.67	0.5026	0	0.11	0.8958
Cat-Mouse	4	2.21	0.0736	0	0.36	0.6977
Cat-Rat	2	1.88	0.2909	0	0.25	0.7788
Cat-Tarsier	3	2.03	0.1483	0	0.32	0.7261
Cat-Colobus	2	0.43	0.0096 **	0	0.16	0.8521
Chicken-Elephant	1	0.19	0.0159 *	0	0.20	0.8187
Chicken-Mouse	0	0.31	0.7334	1	0.22	0.0209 *
Dolphin-Squirrel	1	0.77	0.1805	0	0.13	0.8781
Dolphin-Rat	1	0.96	0.2495	0	0.31	0.7334
Dolphin-Tarsier	2	0.98	0.0767	0	0.28	0.7558
Elephant-Hedgehog	1	1.48	0.5645	0	0.17	0.8437
Elephant-Cat	2	1.54	0.2013	0	0.11	0.8958
Elephant-Pig	2	1.71	0.2454	1	0.11	0.0056 **
Elephant-Cow	1	1.02	0.7284	0	0.28	0.7558
Elephant-Squirrel	1	1.31	0.6233	0	0.03	0.9704
Elephant-Mouse	2	1.64	0.2270	0	0.15	0.8607
Elephant-Rat	2	1.59	0.2141	0	0.12	0.8869
Elephant-Tarsier	1	1.50	0.5578	0	0.19	0.827
Elephant-Marmoset	1	0.31	0.0392 *	0	0.08	0.9231
Hedgehog-Cat	3	2.39	0.2192	0	0.00	N/A
Hedgehog-Pig	5	2.75	0.0608	0	0.00	N/A
Hedgehog-Dolphin	3	0.93	0.0150 *	0	0.32	0.7261
Hedgehog-Cow	2	1.52	0.1962	0	0.38	0.6839
Hedgehog-Squirrel	3	1.58	0.0761	0	0.08	0.9231
Hedgehog-Mouse	5	2.37	0.0339 *	0	0.36	0.6977
Hedgehog-Rat	4	1.91	0.0449 *	0	0.33	0.7189
Hedgehog-Tarsier	3	1.94	0.1322	0	0.29	0.7483
Hedgehog-Colobus	2	0.37	0.0064 **	0	0.21	0.8106
Mallard-Platypus	1	0.47	0.0812	0	0.38	0.6839
Mallard-Mouse	1	0.62	0.1285	0	0.41	0.6637
Mallard-Rat	1	0.40	0.0616	0	0.46	0.6313
Opossum-Elephant	3	1.31	0.0441 *	0	0.24	0.7866
Opossum-Hedgehog	1	1.68	0.4995	0	0.53	0.5886
Opossum-Cat	3	1.67	0.0888	0	0.51	0.6005
Opossum-Pig	3	1.90	0.1253	0	0.54	0.5827
Opossum-Cow	0	1.35	0.2592	1	0.48	0.0842
Opossum-Squirrel	2	1.05	0.0897	0	0.33	0.7189
Opossum-Mouse	4	1.72	0.0309 *	0	0.59	0.5543
Opossum-Rat	5	1.66	0.0072 **	0	0.68	0.5066
Opossum-Tarsier	2	1.74	0.2534	0	0.45	0.6376
Opossum-Marmoset	1	0.35	0.0487 *	0	0.17	0.8437
Platypus-Hedgehog	2	1.15	0.1099	0	0.38	0.6839
Platypus-Pig	2	1.45	0.1787	0	0.45	0.6376
Platypus-Dolphin	1	0.61	0.1252	0	0.36	0.6977
Platypus-Squirrel	1	0.85	0.2093	0	0.33	0.7189
Platypus-Mouse	1	1.51	0.5545	1	0.45	0.0754
Platypus-Rat	2	1.45	0.1787	0	0.47	0.625
Platypus-Tarsier	1	1.19	0.6662	0	0.48	0.6188
Pig-Cow	1	1.83	0.4540	0	0.32	0.7261
Pig-Squirrel	1	1.51	0.5545	0	0.10	0.9048
Pig-Mouse	4	2.36	0.0909	1	0.34	0.0462 *
Pig-Rat	6	2.23	0.0080 **	0	0.35	0.7047
Pig-Tarsier	3	2.29	0.1986	0	0.24	0.7866
Rat-Tarsier	5	1.87	0.0123 *	0	0.36	0.6977
Squirrel-Mouse	1	1.53	0.5478	0	0.13	0.8781
Squirrel-Tarsier	1	1.48	0.5645	0	0.20	0.8187
Squirrel-Marmoset	1	0.34	0.0462 *	0	0.10	0.9048
Mouse-Tarsier	3	2.23	0.1866	0	0.34	0.7118
Mouse-Marmoset	1	0.37	0.0537	0	0.22	0.8025
Mouse-Colobus	1	0.39	0.0589	0	0.18	0.8353
Tarsier-Colobus	1	0.42	0.0670	0	0.16	0.8521

## Supplementary Materials

The following are available online at https://www.mdpi.com/2073-4425/10/3/181/s1, Table S1: Details of supported programs in *ProtParCon*.
