# Supplementary material for: ProtParCon: A Framework for Processing Molecular Data and Identifying Parallel and Convergent Amino Acid Replacements
Source: Genes (Basel). 2019 Feb 26;10(3):181. doi: 10.3390/genes10030181 (PMC6471834; doi:10.3390/genes10030181)
Supplement: Supplementary file 1 [file genes-10-00181-s001.pdf]

Table 1. Details of supported programs in *ProtParCon*.

Programs	Version[1]	Downloadable Address	Installation Instruction
MUSCLE	3.8.31	https://www.drive5.com/muscle/downloads.htm	https://www.drive5.com/muscle/manual/install.html
MAFFT	7.402	https://mafft.cbrc.jp/alignment/software/	https://mafft.cbrc.jp/alignment/software/
Clustal Omega	1.2.4	http://www.clustal.org/omega/	http://www.clustal.org/omega/INSTALL
T-COFFEE	12.00	http://www.tcoffee.org/Projects/tcoffee/index.html	http://www.tcoffee.org/Projects/tcoffee/index.html
PAML	4.9	http://abacus.gene.ucl.ac.uk/software/paml.html	http://abacus.gene.ucl.ac.uk/software/paml.html
RAxML	8.2.12	https://github.com/stamatak/standard-RAxML	https://cme.h-its.org/exelixis/web/software/raxml/hands_on.html
FastTree	2.1	http://www.microbesonline.org/fasttree/	http://www.microbesonline.org/fasttree/
IQ-TREE	1.6.6	http://www.iqtree.org/#download	http://www.iqtree.org/doc/Quickstart#installation
PhyML	3.0	http://www.atgc-montpellier.fr/phyml/binaries.php	http://www.atgc-montpellier.fr/phyml/
Seq-Gen	1.3.4	http://tree.bio.ed.ac.uk/software/seqgen/	http://tree.bio.ed.ac.uk/software/seqgen/

[1] The version numbers denoted in the table showing the version of the corresponding program has been tested. Users are recommended to use the latest version.
